# Dynamically Tunable Plasmon-Induced Transparency in On-chip Graphene-Based Asymmetrical Nanocavity-Coupled Waveguide System

**DOI:** 10.1186/s11671-017-2148-z

**Published:** 2017-05-25

**Authors:** Pingping Qiu, Weibin Qiu, Zhili Lin, Houbo Chen, Junbo Ren, Jia-Xian Wang, Qiang Kan, Jiao-Qing Pan

**Affiliations:** 1 Fujian Key Laboratory of Light Propagation and Transformation, College of Information Science and Engineering, Huaqiao University, Xiamen, 361021 China; 2 Institute of Semiconductors, Chinese Academy of Sciences, 35A, Qinghua East Road, Haidian District, Beijing, 100086 China

**Keywords:** Graphene, Plasmon-induced transparency, Surface plasmon polaritons, Refractive index sensor

## Abstract

A graphene-based on-chip plasmonic nanostructure composed of a plasmonic bus waveguide side-coupled with a U-shaped and a rectangular nanocavities has been proposed and modeled by using the finite element method in this paper. The dynamic tunability of the plasmon-induced transparency (PIT) windows has been investigated. The results reveal that the PIT effects can be tuned via modifying the chemical potential of the nanocavities and plasmonic bus waveguide or by varying the geometrical parameters including the location and width of the rectangular nanocavity. Further, the proposed plasmonic nanostructure can be used as a plasmonic refractive index sensor with a sensing sensibility of 333.3 nm/refractive index unit (RIU) at the the PIT transmission peak. Slow light effect is also realized in the PIT system. The proposed nanostructure may pave a new way towards the realization of graphene-based on-chip integrated nanophotonic devices.

## Background

Plasmon-induced transparency (PIT), which is a novel phenomenon analogous to electromagnetically induced transparency (EIT) effect generating a sharp transparency window within a broad absorption spectrum [1], has attracted great attention due to its potential applications in a wide range of fields, such as slow light [2, 3], optical switching [4], light storage [5], and high-sensitivity sensing [6, 7]. The PIT-based devices can be realized with ultra-compact footprint because of the large local field enhancement ability and overcoming of classical diffraction limit of light provided by the surface plasmon polaritons (SPPs) [8, 9]. A variety of designs have been proposed to achieve PIT effect in plasmonic nanostructures including coupled resonator systems [10–13], photonic crystal structures [14, 15], and metamaterial structures [16, 17]. However, most of these structures displaying PIT effect are barely tunable unless changing the geometrical parameters of the structures, which in a large extent limit the active control of PIT windows and degrade the quality.

Graphene, a monolayer of carbon atoms arranged in a two-dimensional (2D) honeycomb lattice [18], shows great potential for developing highly efficient optoelectronic devices due to its exceptional electrical and optical properties including the ability of extreme confinement [19–21], dynamic tunability, and relatively low damping losses [22, 23]. Particularly, the surface conductivity of graphene can be dynamically tuned by chemical potential via external gate voltage or chemical doping [24, 25], which makes graphene to be a promising candidate for designing tunable PIT while the geometrical parameters are fixed. Because of these extraordinary features compared to those of conventional noble metals, a wide range of researches have been done to realize graphene-based PIT, such as PIT phenomena in graphene ring resonator-coupled graphene waveguide [26, 27] and PIT effects in a graphene-based nanoribbon waveguide coupled with graphene rectangular resonator structure [28, 29]. Sun et al. studied the periodically patterned graphene double-layer structure separated by a dielectric layer in the terahertz frequency range, where the multispectral PIT responses have been achieved [30]. Furthermore, tunable PIT effects are realized in the periodically combined graphene nanostrips and analytically described with the coupled Lorentz oscillator model [31, 32]. However, most of the previous works were concerned about graphene resonators, coupled to a monolayer graphene or a graphene nanoribbon waveguide system, and graphene nanostrip systems with normal incident light. There were very few or even no studies about plasmonically induced transparency phenomenon in a graphene sheet with locally variant chemical potentials. Further, compared with normal incident light, in plane propagation has overwhelming advantages for on-chip integration.

Motivated by the above fundamental studies, in this paper, we propose a graphene-based plasmonic nanostructure composed of a plasmonic bus waveguide side-coupled to a U-shaped nanocavity and a rectangular nanocavity on the same graphene monolayer. The commercial software COMSOL Multiphysics based on finite element method (FEM) is utilized to explore the transmission and electromagnetic responses of our designs. Simulation results reveal that the PIT phenomenon is observed in our proposed plasmonic nanostructure. Further, the PIT window can be effectively tuned by varying the chemical potentials of the nanocavities and plasmonic bus waveguide. Also, a coupled mode theory (CMT) is introduced to explain the transmission features of the PIT phenomenon. At last, a plasmonic refractive index sensor based on the proposed plasmonic nanostructure is studied. The sensing sensitivity of 333.3 nm/refractive index unit (RIU) is achieved at the PIT transmission peak. Also, the slow light effect with group delay over 1 ps is realized. This proposed novel plasmonic nanostructure may offer a new way to realize graphene-based on-chip high-density plasmonic device integration on a graphene monolayer.

## Methods

**Fig. 1:**
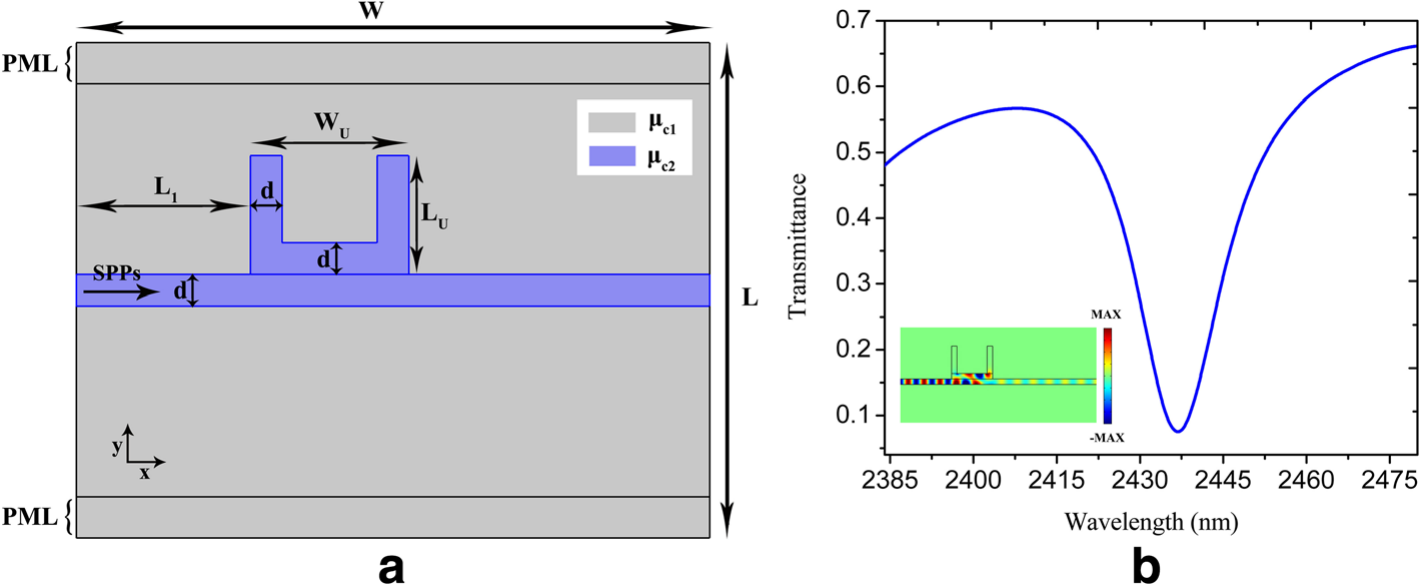
**a, b** The schematic configuration and geometric parameters of a U-shaped nanocavity-coupled waveguide system and the corresponding spectral transmittance respectively. The *inset* in **b** shows the electric field (*E*_y_) distribution at a wavelength of 2437 nm. The parameters are set as *W* = 800 nm, *L* = 620 nm, *d* = 20 nm, *W*_U_ = 150 nm, *L*_U_ = 120 nm, *L*_1_ = 220 nm, *τ* = 1 ps, *μ*_c1_ = 0.3 eV, and *μ*_c2_ = 0.9 eV. The perfectly matched layers (*PML*) with a width of 50 nm are implemented on the *top* and *bottom* of the computation domain to eliminate undesired reflections from the domain boundary

For the sake of simplicity, the proposed structure is modeled by a suspended graphene monolayer with local variation of chemical potential to form the corresponding bus waveguide and the nanoresonators. Figure 1a shows the schematic configuration and geometric parameters of a U-shaped nanocavity directly coupled to a plasmonic bus waveguide. The U-shaped nanocavity coupled waveguide with a chemical potential of *μ*_c2_ is surrounded by the same sheet of graphene with chemical potential of *μ*_c1_. The width of the plasmonic bus waveguide d is 20 nm. The width and height of the U-shaped nanocavity are *W*_U_ = 150 nm and *L*_U_ = 120 nm respectively. Exact theoretical modeling of such a structure requires a three-dimensional (3D) computation, which is extremely time- and memory-consuming. To solve this problem, the effective index method has been used by many publications [33–35], and the refractive index of the structure is replaced by the effective index of guided modes, which is defined by the ratio between the propagation constant and the wave number in the free space. In our structure, the graphene sheet is treated as an ultrathin film which is characterized by effective index defined as *n*_eff_ = *β*/*k*_0_, where *k*_0_ = 2*π*/*λ* is the wave number in free space. The propagation constant *β* of the guided SPP mode supported by monolayer graphene is written as [36, 37]

1 $$ \beta ={k}_0\sqrt{1-{\left(\frac{2}{\sigma_{\mathrm{g}}\sqrt{\mu_0{\mu}_{\mathrm{r}}/{\varepsilon}_0{\varepsilon}_{\mathrm{r}}}}\right)}^2,} $$

where *μ*_0_ and *ε*_0_ represent the permeability and permittivity of vacuum, respectively, and *μ*_r_ and *ε*_r_ represent the relative permeability and relative permittivity respectively. The surface conductivity of graphene *σ*_g_ composed of the interband electron transitions *σ*_inter_ and the intraband electron-photon scattering *σ*_intra_ is given by the Kubo formula [38, 39]

2 $$ {\sigma}_{\mathrm{g}}={\sigma}_{\mathrm{intra}}+{\sigma}_{\mathrm{inter}} $$

With

3 $$ {\sigma}_{\mathrm{intra}}=\frac{- i{e}^2{k}_{\mathrm{B}} T}{\pi {\hslash}^2\left(\omega - i/\tau \right)}\left[\frac{\mu_{\mathrm{c}}}{k_{\mathrm{B}} T}+2 \ln \left(1+ \exp \left(-\frac{\mu_{\mathrm{c}}}{k_{\mathrm{B}} T}\right)\right)\right] $$

4 $$ {\sigma}_{\mathrm{inter}}=\frac{- i{e}^2}{2 h} \ln \left[\frac{2\left|{\mu}_c\left|-\hslash \left(\omega - i/\tau \right)\right.\right.}{2\left|{\mu}_c\left|+\hslash \left(\omega - i/\tau \right)\right.\right.}\right] $$

where *μ*_c_ is the chemical potential of graphene, *ω* is the angular frequency of the plasmon, *ћ* is the reduced Planck’s constant, *e* is the electron charge, *k*_B_ is the Boltzmann’s constant, *T* is the temperature, ℏ = h/2*π* is the reduced Planck’s constant, and *τ* is the electron momentum relaxation time. Specifically, the chemical potential of graphene can be tuned via chemical doping or electrical gating [25, 26]. Mikhailov et al. have experimentally shown that the carrier density in a graphene sheet as high as 10^14^ cm^−2^ had been achieved, which led to a chemical potential of 1–2 eV at a temperature below 250 K [40]. Furthermore, it has been demonstrated that high-quality suspended graphene with direct current mobility as high as 10^5^ cm^2^V^−1^s^−1^ can be obtained, which corresponds to *τ* > 1.5 ps [41]. In this paper, both the relaxation time and chemical potential we set are conservative enough to ensure the reliability of our numerical study.

## Results and Discussion

**Fig. 2:**
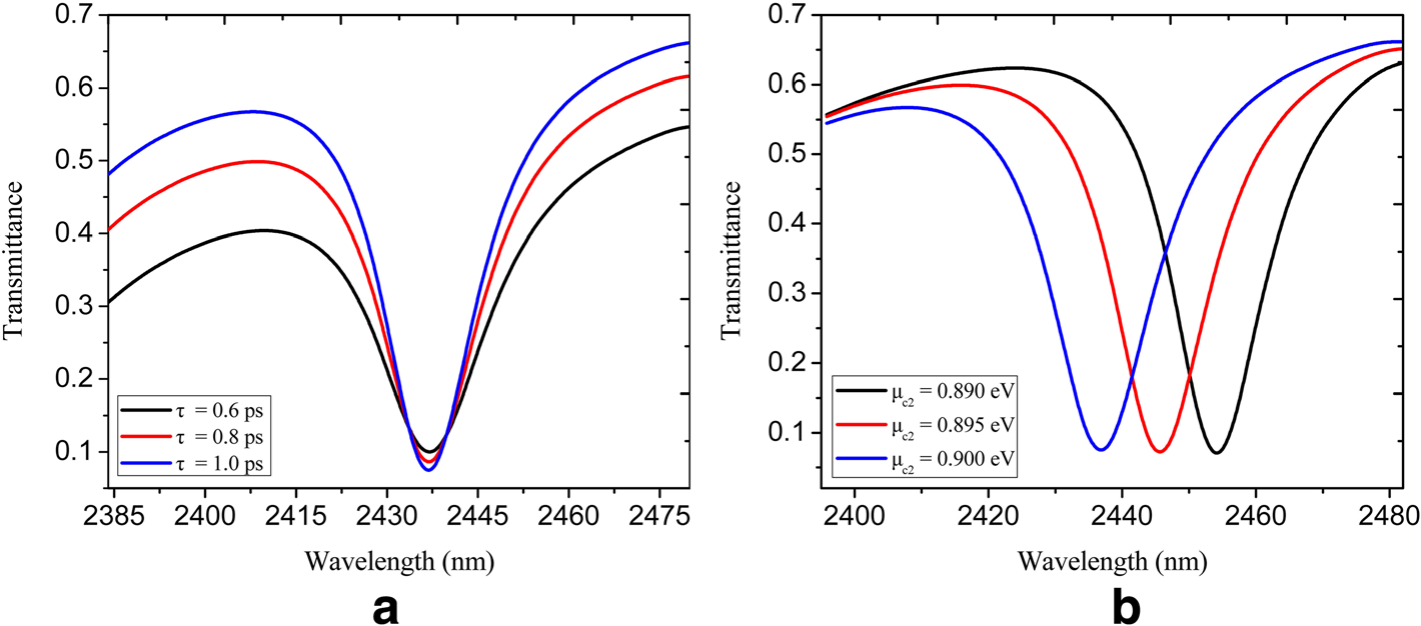
Spectral transmittance of a U-shaped nanocavity-coupled waveguide system shown in Fig. 1: **a** with *τ* = 0.6, 0.8, and 1 ps; *μ*_c1_ = 0.3 eV; and *μ*_c2_ = 0.9 eV; **b** with *μ*_c2_ = 0.89, 0.895, and 0.9 eV; *μ*_c1_ = 0.3 eV; and *τ* = 1 ps

As the SPP wave passes through the side-coupled U-shaped nanocavity shown in Fig. 1a, the energy is coupled into the nanocavity. A deep transmission valley is obtained at the resonance wavelength due to the destructive interference between the incident wave and escaped power from the nanocavity [12, 13]. Figure 1b plots the transmission spectrum of a U-shaped nanocavity directly coupled to a plasmonic bus waveguide with *τ* = 1 ps, *μ*_c1_ = 0.3 eV, and *μ*_c2_ = 0.9 eV. A pronounced dip with transmittance less than 0.1 is achieved at the resonance wavelength of 2437 nm. The inset in Fig. 1b shows the corresponding electric field distribution at the resonance wavelength, where it can be seen that almost no SPPs propagate through the plasmonic waveguide. Figure 2a displays the transmittance spectra with varied relaxation time *τ* = 0.6, 0.8, and 1 ps, where it can be seen that a higher transmission contrast is achieved when the relaxation time increases. This is attributed to the reduction of the Ohmic absorption of the plasmons when the relaxation time of electron momentum increases [39]. The calculated transmittance of a U-shaped nanocavity-coupled waveguide system for different chemical potentials *μ*_c2_ is presented in Fig. 2b. The relaxation time *τ* and chemical potential *μ*_c1_ are constantly kept as 1 ps and 0.3 eV respectively. One can see that the locations of dips are dynamically tuned via varied chemical potential of the nanocavity and bus waveguide. The central wavelengths of the dips are 2455, 2445, and 2437 nm with *μ*_c2_ = 0.89, 0.895, and 0.9 eV respectively.

According to CMT [12, 42, 43], the spectral transmittance of the system supporting a resonant mode of frequency *ω*_0_ can be written as

5 $$ T=\frac{{\left(\omega -{\omega}_0\right)}^2+{\left(1/{\tau}_i\right)}^2}{{\left(\omega -{\omega}_0\right)}^2+{\left(1/{\tau}_i+1/{\tau}_e\right)}^2} $$

where 1/*τ*_i_ and 1/*τ*_e_ represent the decay rate of the intrinsic loss in the nanocavity and the power escaping through the plasmonic bus waveguide respectively. Obviously, the minimum transmittance *T*_min_ = (1/*τ*_i_)^2^/(1/*τ*_i_ + 1/*τ*_e_)^2^ can be achieved when the frequency of incident light *ω* is equal to the resonance frequency *ω*_0_. As the 1/*τ*_e_ is much more than 1/*τ*_*i*_, a transmission dip nearly zero can be obtained, which agrees well with the simulation results.

**Fig. 3:**
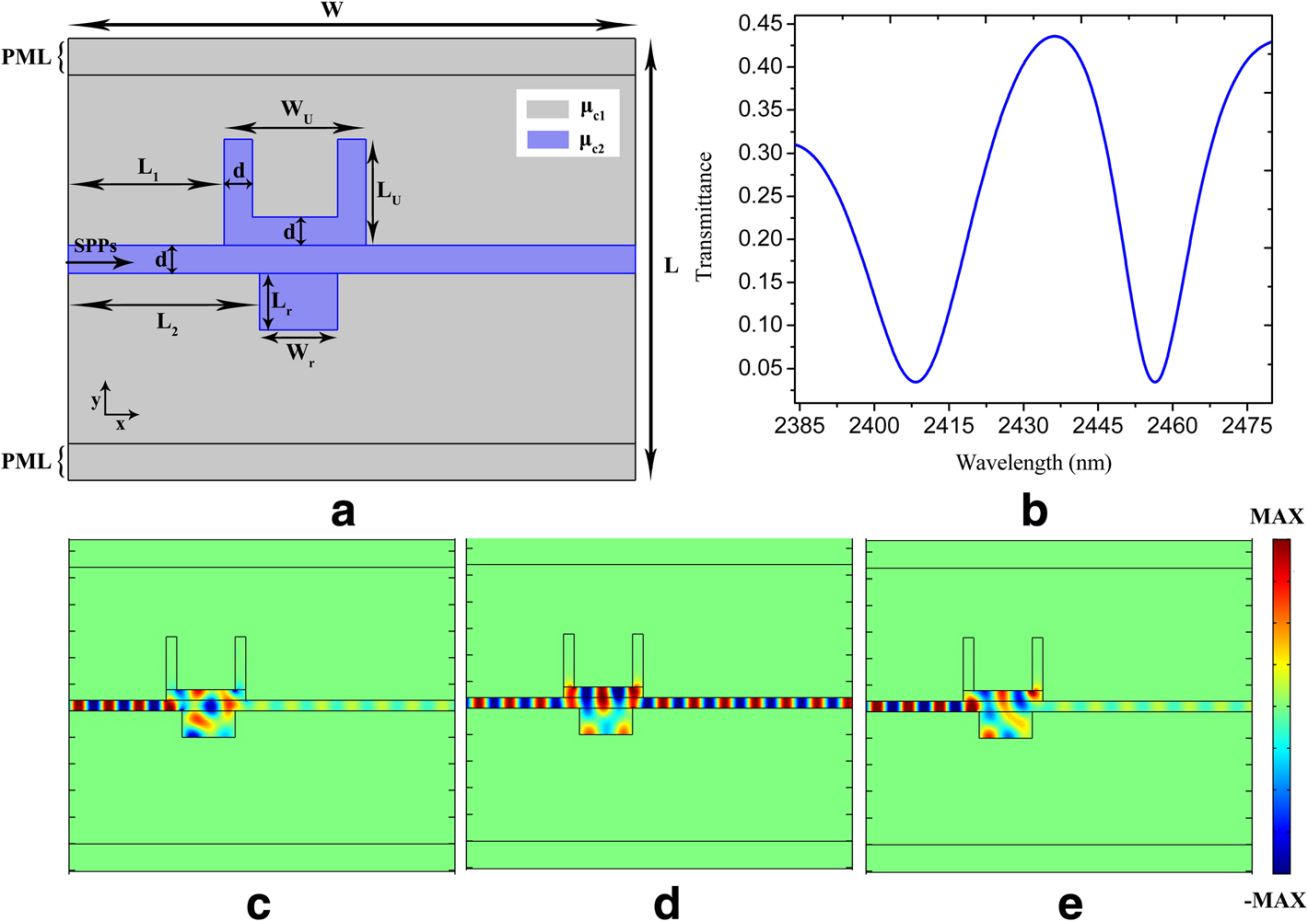
**a, b** The schematic configuration and the geometric parameters of U-shaped and rectangular nanocavity-coupled waveguide system and the corresponding spectral transmittance respectively. **c**–**e** Electric field (*E*_y_) distribution at wavelengths of 2408, 2437, and 2457 nm respectively. The parameters are set as *W* = 800 nm, *L* = 620 nm, *d* = 20 nm, *W*_U_ = 150 nm, *L*_U_ = 120 nm, *L*_1_ = 220 nm, *L*_2_ = 250 nm, *L*_r_ = 50 nm, *W*_*r*_ = 100 nm, *τ* = 1 ps, *μ*_c1_ = 0.3 eV, and *μ*_c2_ = 0.9 eV

In order to obtain PIT effects, we add a rectangular nanocavity based on the plasmonic nanostructure shown in Fig. 1. A graphene-based plasmonic nanostructure composed of a plasmonic bus waveguide side-coupled to U-shaped and rectangular nanocavities is schematically shown in Fig. 3a. There exists strong coupling between the two nanocavities when they are connected via the plasmonic bus waveguide. The destructive interference between two resonant excitation pathways related to the U-shaped and rectangular nanocavities generates the PIT phenomenon [10, 11]. As shown in Fig. 3b, a sharp transmission peak (increased from 0.06 to 0.44) appeared in the transmission forbidden band shown in Fig. 1b implying the formation of the PIT window. The central wavelength of the PIT window is 2437 nm which is exactly the location of the central wavelength of the transmission dip shown in Fig. 1b. The broad resonant of the U-shaped nanocavity is split into two resonant modes: one is blueshifted while the other is redshifted [12, 13]. Figure 3c–e displays the electric field distributions of resonant modes at 2408, 2437, and 2457 nm respectively. We can see that the electric field distribution in the nanocavities is in-phase with the electric field distribution in plasmonic bus waveguides at 2437 nm, which means that the incident light and the light escaping into the plasmonic bus waveguide from the nanocavities encounter a coherent enhancement. Furthermore, the electric field distributions reveal that there is anti-phase between the nanocavities and plasmonic bus waveguide at 2408 and 2457 nm, i.e., the conditions of destructive resonance have been met which results in the inhibition of the transmission waves [12].

**Fig. 4:**
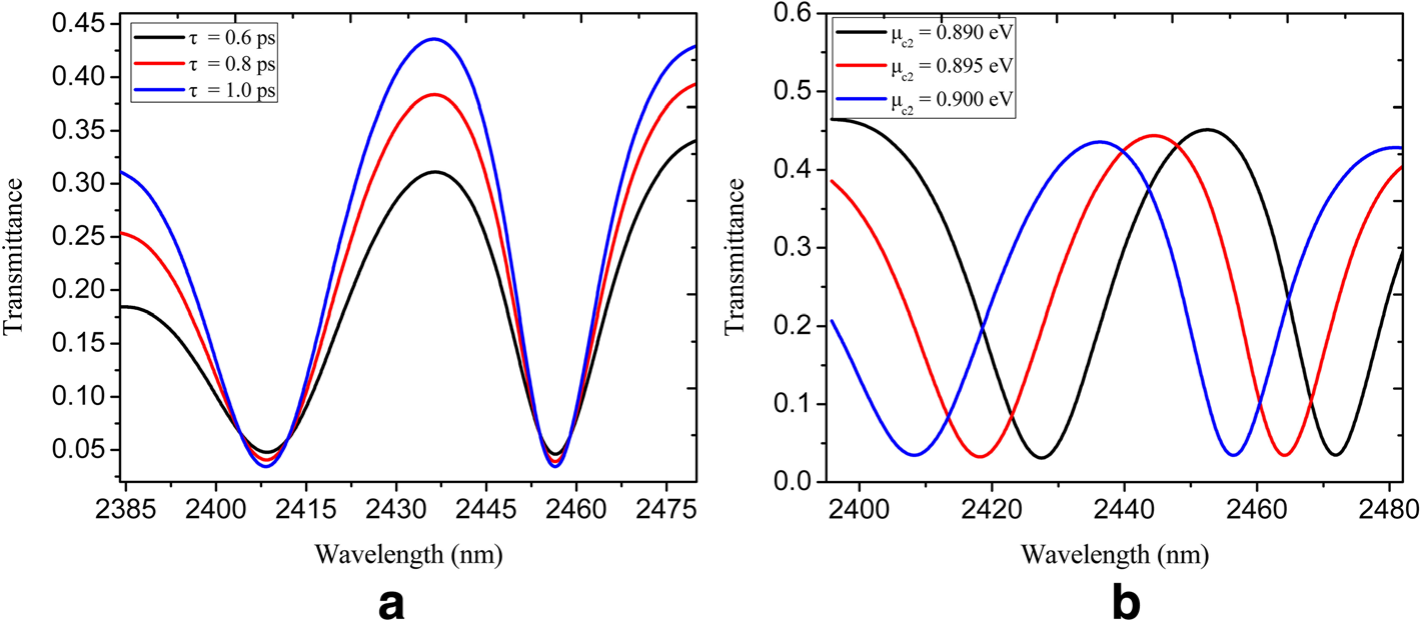
The spectral transmittance of U-shaped and rectangular nanocavity-coupled waveguide system shown in Fig. 3: **a** with *τ* = 0.6, 0.8, and 1 ps; **b** with *μ*_c2_ = 0.89, 0.895, and 0.9 eV

We calculate the spectral transmittance for the U-shaped and rectangular nanocavity-coupled plasmonic bus waveguide system with varied relaxation time *τ* = 0.6, 0.8, and 1 ps, and the results are shown in Fig. 4a. One can see that the transmission contrast increases with the increasing of relaxation time. Moreover, the dynamic tunability of the PIT window is shown in Fig. 4b. The chemical potential *μ*_c1_ is constantly kept as 0.3 eV, while *μ*_c2_ is 0.89, 0.895, and 0.9 eV. As the chemical potential *μ*_c2_ increases, the transmission peak (at the wavelengths of 2452, 2445, and 2437 nm) in the PIT window is obviously blueshifted. As a result, the dynamically tunable PIT effect is realized in our proposed nanostructure by modifying the chemical potential of the nanocavities and plasmonic bus waveguide.

**Fig. 5:**
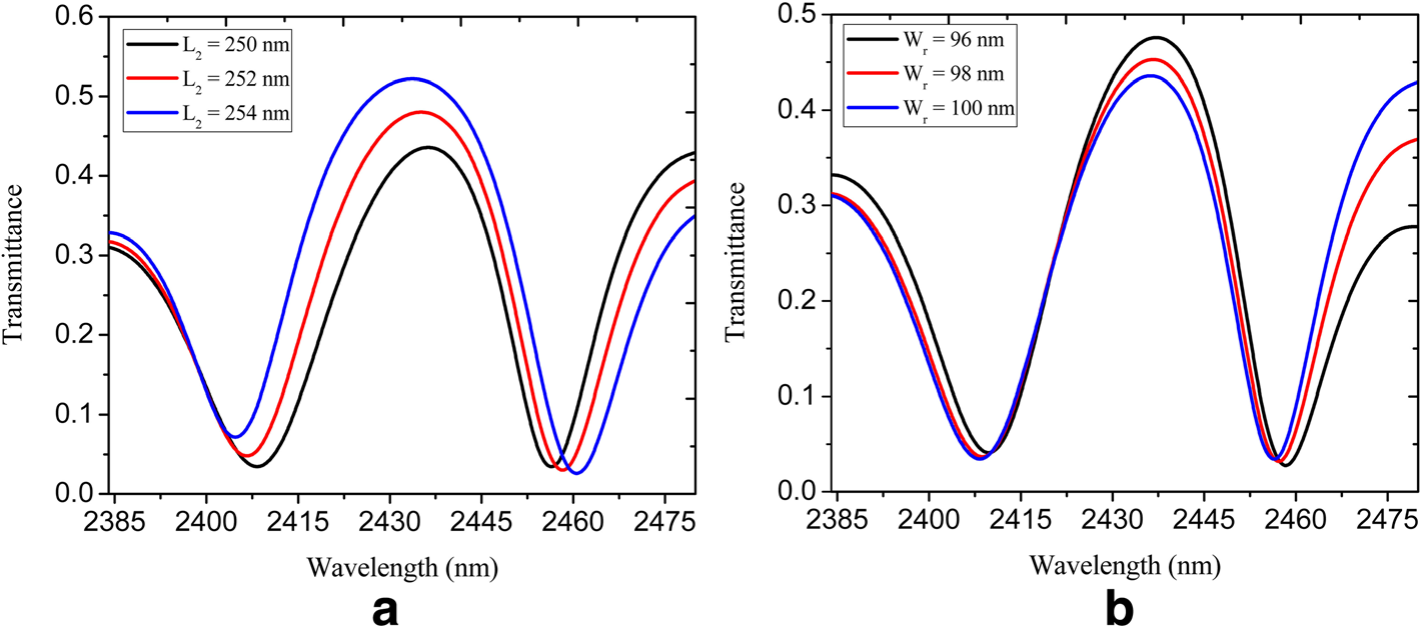
Spectral transmittance of U-shaped and rectangular nanocavity-coupled waveguide system shown in Fig. 3: **a** with *L*_2_ = 250, 252, and 254 nm; **b** with *W*_r_ = 96, 98, and 100 nm

To investigate how the geometrical parameters influence the PIT phenomenon, we modified the location of the rectangular nanocavity. Figure 5a shows the spectral transmittance of the U-shaped and rectangular nanocavity-coupled plasmonic bus waveguide system, where it is seen that the transmission peak got higher (increased from 0.44 to 0.52) and the PIT window becomes broader with *L*_2_ increasing for a certain range, which is attributed to the intensification of the coupling strength between the two nanocavities [11, 28]. Also, we find that the decreasing of the width of rectangular nanocavity can lead to a higher transmission peak (increased from 0.44 to 0.48) as shown in Fig. 5b. This offers another option to tune the PIT window. The quality factor (Q-factor) of the PIT windows is defined as *λ*_*0*_/∆*λ*, where *λ*_0_ and ∆*λ* are transmission peak wavelength and full width at half maximum (FWHM). In our proposed plasmonic nanostructure, a FWHM of less than 30 nm and Q-factor of around 80 is obtained, which is much narrower and higher than the counterparts of graphene-based PIT proposed in the aforementioned references [28, 29].

In accordance with CMT, the transmittance in our plasmonic nanostructure is expressed as [12, 42]

6 $$ T={\left|\frac{j\left({\omega}_{\mathrm{U}}-{\omega}_{\mathrm{r}}\right)+\gamma +1}{j\left({\omega}_{\mathrm{U}}-{\omega}_{\mathrm{r}}\right)+\beta +\gamma +1}\right|}^2 $$

where *γ* and *β* stand for the coupling coefficient between the two nanocavities and the coupling coefficient between the nanocavities and plasmonic bus waveguide respectively. We can find that the PIT window can be obtained when the resonant frequencies of the U-shaped nanocavity *ω*_U_ and the rectangular nanocavity *ω*_r_ are approximately equivalent. And the corresponding transmission peak is |( *γ* + 1)/(*β* + *γ* + 1)|^2^.

**Fig. 6:**
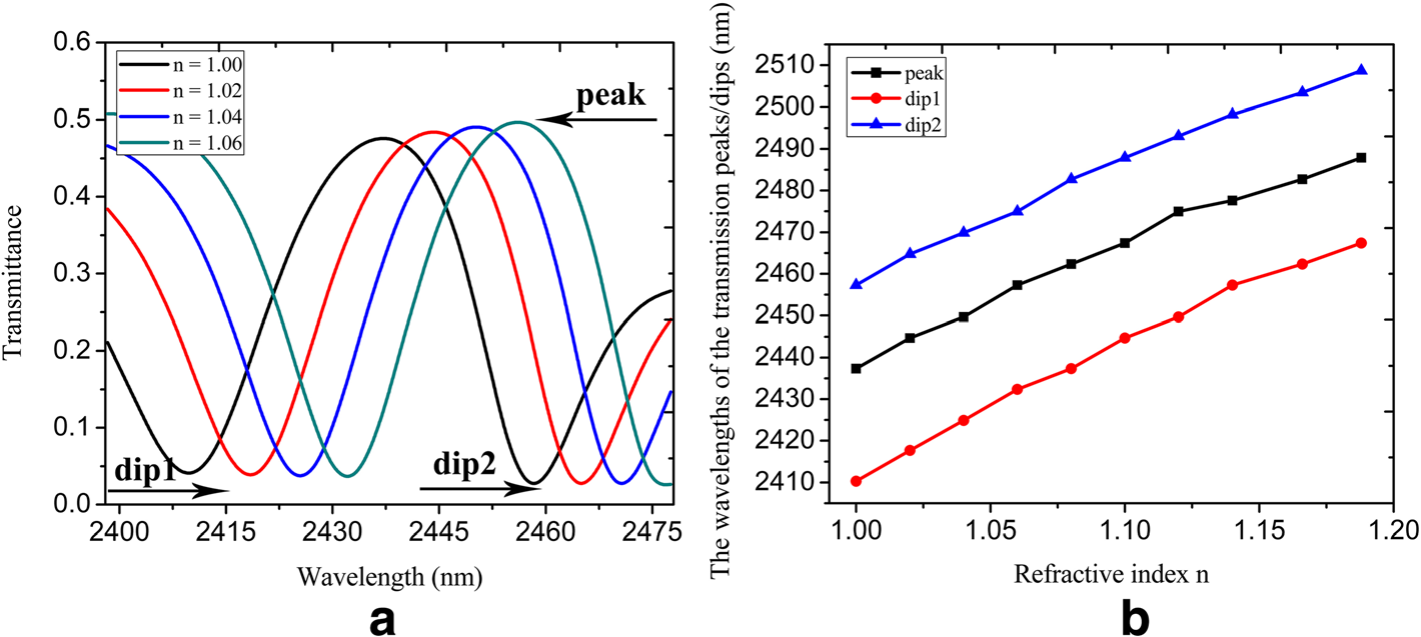
**a** The spectral transmittance with refractive index *n* = 1, 1.02, 1.04, and 1.06; **b** the peak/dip wavelengths of the spectral transmittance versus the refractive index *n*. The parameters are set as *W* = 800 nm, *L* = 620 nm, *d* = 20 nm, *W*_U_ = 150 nm, *L*_U_ = 120 nm, *L*_1_ = 220 nm, *L*_2_ = 250 nm, *L*_r_ = 50 nm, *W*_r_ = 96 nm, *τ* = 1 ps, *μ*_c1_ = 0.3 eV, and *μ*_c2_ = 0.9 eV

Based on the structure shown in Fig. 3a, we construct the refractive index sensor, which is realized by modifying the relative permittivity in Eq. 1. Figure 6a illustrates the spectral transmittance with different refractive index *n*, which refers to the refractive index of the undersensing material. One can see that the peak/dip1/dip2 wavelengths shift from 2437.3 to 2457.3 nm/2410.3 to 2432.4 nm/2457.3 to 2474.9 nm when the refractive index *n* varies from 1 to 1.06. As the refractive index *n* increases, both the transmission peak and dips exhibit a redshift. The sensing sensitivity of refractive index sensor, defined as the shift in the peak/dip1/dip2 wavelength per unit variations of the refractive index *dλ/dn* is 333.3, 368.3, and 293.3 nm/RIU respectively. Figure 6b shows the peaks and dips of the spectral transmittance with refractive index *n* varying from 1 to 1.19, where we can see the approximately linear relationship of the peak/dip wavelengths versus the refractive index *n*.

**Fig. 7:**
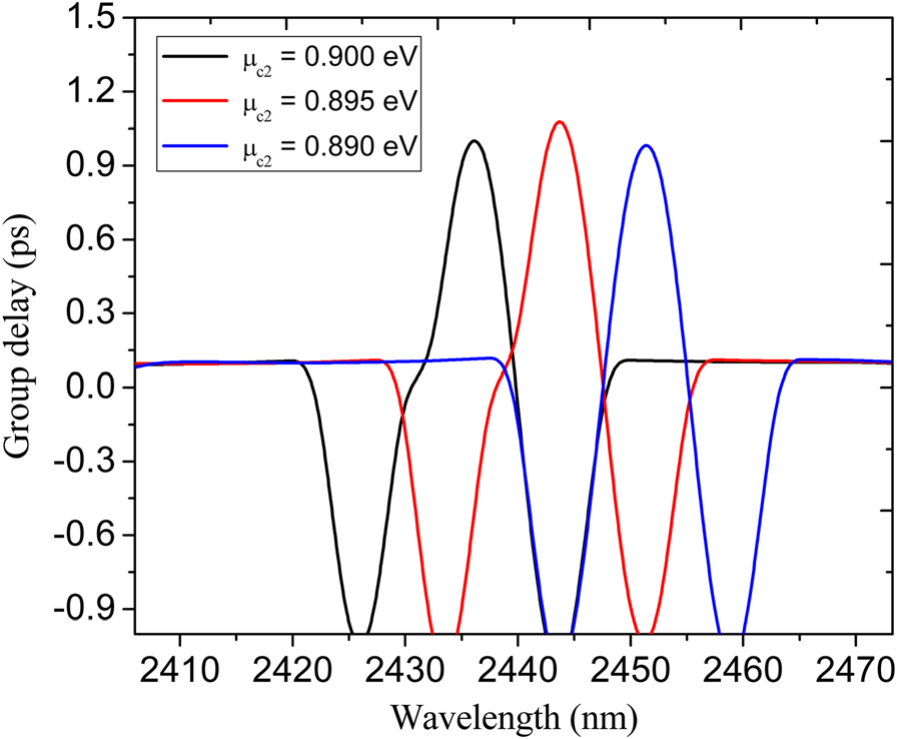
The group delays versus chemical potential *μ*_c2_ for the graphene PIT system shown in Fig. 3a. The other parameters are set as *W* = 800 nm, *L* = 620 nm, *d* = 20 nm, *W*_U_ = 150 nm, *L*_U_ = 120 nm, *L*_1_ = 220 nm, *L*_2_ = 254 nm, *L*_r_ = 50 nm, *W*_*r*_ = 96 nm, *τ* = 1 ps, *μ*_c1_ = 0.3 eV

It is well known that the PIT phenomenon is accompanied by the slow light effect caused by the sharp dispersion [13, 29]. The slow light effect can be characterized by the group delay expressed as *τ*_g_ = *∂φ*(*ω*)/∂ *ω* where *φ*(*ω*) is the effective phase shift of the transmission spectrum. In Fig. 7, we plot the group delays within the PIT window at different chemical potential *μ*_c2_. In the vicinity of the PIT transmission peak, it offers large positive group delays indicating the slow light effect. The peak wavelengths of the PIT system at *μ*_c2_ = 0.89, 0.895, and 0.9 eV are 2449.7, 2442.3, and 2434.7 nm, respectively, and the corresponding group delays are 0.99, 1.1, and 1.02 ps respectively. Thus, the slow light effect is effectively tuned by modifying the chemical potential of the nanocavities and the plasmonic bus waveguide. It should also been pointed out that this is a proof-of-concept article. In reality, the proposed structure should lie on the substrate, where the refractive index is larger than the air, and the frequency response would shift accordingly. Also, the confinement of the plasmon is higher, accompanying by the increasing of the loss, which results in the reduction of the peak value of the transparence window in the transmission spectrum. However, the principle is identical to the suspended case.

## Conclusions

In conclusion, dynamically tunable PIT effects in graphene-based plasmonic nanostructure composed of a plasmonic bus waveguide side-coupled to U-shaped and rectangular nanocavities have been proposed and modeled by using finite element method. The dynamic tunability of the PIT windows is obtained by modifying the chemical potential of the nanocavities and plasmonic bus waveguide. Furthermore, the PIT window can be tuned dynamically via adjusting the geometrical parameters of the nanostructure, such as the location and width of the rectangular nanocavity. Compared to the conventional ring resonators [24, 25], our proposed asymmetric U-shaped and rectangular resonators offer stronger coupling strength between the resonators and the bus waveguide, which further results in the stronger PIT effect. On the other hand, unlike other reported nanoribbon waveguides, our structures are formed by the local variation of chemical potential on the identical graphene monolayer, and this provides the easier integration with other functional components on the same material platform. Moreover, this plasmonic nanostructure can be used as refractive index sensor with high sensing sensibility. And the slow light effect with a large group delay is also realized in the PIT system. The proposed nanostructure paves a new way towards the realization of graphene-based on-chip integrated nanophotonic devices.

## Abbreviations

CMT: Coupled mode theory
EIT: Electromagnetically induced transparency
FEM: Finite element method
PIT: Plasmon-induced transparency
RIU: Refractive index unit
SPPs: Surface plasmon polaritons
